# Clinical Profile, Endoscopic Findings, and Outcomes Among Inpatients With Upper Gastrointestinal Bleeding in a United Kingdom District General Hospital

**DOI:** 10.7759/cureus.97644

**Published:** 2025-11-24

**Authors:** Mansur F Mohammed, Rajesh K Gurunathan, Nourhan N Eltanikhy, Pavithra Dhanasekaran

**Affiliations:** 1 Gastroenterology, University Hospitals of Derby and Burton NHS Foundation Trust, Burton-on-Trent, GBR; 2 Acute Internal Medicine, University Hospitals of Derby and Burton NHS Foundation Trust, Burton-on-Trent, GBR

**Keywords:** acute upper gastrointestinal bleeding, endoscopy, gastrointestinal bleeding, gastrointestinal hemorrhage, hematemesis, non-variceal bleeding, risk stratification, upper gi bleed, variceal bleeding

## Abstract

Background

Acute upper gastrointestinal bleeding (AUGIB) is a medical emergency associated with significant morbidity and mortality. Prognosis and management are typically determined by the patient’s clinical features and findings on endoscopy.

Objective

This study aimed to evaluate the demographic characteristics, risk stratification, endoscopic findings, and therapeutic interventions in patients presenting with AUGIB at Queens Hospital, Burton, United Kingdom, in 2024.

Methods

A retrospective analysis was performed of all inpatients who underwent upper gastrointestinal endoscopy for hematemesis and/or melena from January to December 2024. Data on demographics, Glasgow-Blatchford Score (GBS), hemodynamic status, endoscopic diagnoses, interventions, and outcomes were obtained from electronic medical records and analyzed using SPSS Version 23 (IBM Corp., Armonk, NY, USA).

Results

Sixty-four patients (mean age 72.1±15.8; 71% male) were included. Nonvariceal bleeding accounted for 92% of cases, with duodenal ulcers (17%) and gastric ulcers (11%) as leading etiologies. All patients were risk-assessed using the GBS on admission. Twenty-five percent of patients with nonvariceal bleeding required therapeutic endoscopy, primarily using dual or triple modalities. Variceal bleeds (8%) were managed with terlipressin, antibiotics, and band ligation based on endoscopic grade. Re-bleeding occurred in 3.1% of patients, necessitating further interventions.

Conclusion

AUGIB predominantly affected elderly males, with nonvariceal etiologies, particularly peptic ulcers, being most common. Universal risk stratification and timely endoscopic intervention in line with best practices helped improve outcomes. The study recorded a low re-bleeding rate.

## Introduction

Acute upper gastrointestinal bleeding (AUGIB) is among the most frequent emergencies encountered in both gastroenterology and general medicine, requiring prompt evaluation and management to reduce morbidity and mortality [1]. It results from bleeding within the esophagus, stomach, or duodenum and typically presents as hematemesis, coffee-ground vomiting, and/or melena [1]. Occasionally, brisk upper gastrointestinal (GI) bleeding may present as hematochezia [2].

The global incidence of AUGIB is estimated to range between 50 and 150 cases per 100,000 individuals each year, with variations influenced by demographic factors and underlying comorbidities [2]. In the United Kingdom, epidemiological data suggest that there are up to 172 hospital admissions per 100,000 population annually, highlighting the significant burden on healthcare resources [3]. Despite advances in diagnosis and treatment, inpatient mortality remains approximately 7-10%, emphasizing its ongoing clinical significance [1,2].

Risk factors for AUGIB include Helicobacter pylori infection, use of nonsteroidal anti-inflammatory drugs (NSAIDs), antiplatelet or anticoagulant therapy, excessive alcohol consumption, and the presence of comorbid illnesses [4,5]. The causes of AUGIB are broadly classified as nonvariceal or variceal [4]. Nonvariceal bleeding, most often due to peptic ulcer disease, erosive esophagitis, or vascular lesions, accounts for most cases in developed countries [4]. In contrast, variceal bleeding, which usually results from portal hypertension secondary to cirrhosis, is less common but carries a considerably higher risk of mortality [6].

Endoscopy remains the cornerstone for diagnosing the source of bleeding, assessing risk, and delivering definitive therapy [5,7,8]. Advances in endoscopic techniques, including thermal coagulation, clip application, and hemostatic sprays, have contributed to substantial improvements in clinical outcomes, either when used alone or in combination [5,7,8]. Current guidelines and consensus statements from the British Society of Gastroenterology (BSG), the European Society of Gastrointestinal Endoscopy (ESGE), and the American College of Gastroenterology (ACG) recommend performing endoscopy within 24 hours of presentation, with urgent procedures reserved for patients who remain hemodynamically unstable despite initial resuscitation [7,9,10].

Incorporating risk assessment tools into clinical practice is now a key component of guideline-based management [5,7]. Risk scoring systems such as the Glasgow-Blatchford Score (GBS) and the Rockall score assist clinicians in triaging patients, determining the required level of care, predicting the likelihood of therapeutic intervention, and possible outcomes [5,11]. Among these systems, the GBS has been validated across a range of populations and healthcare settings, demonstrating reliable predictive accuracy for both the need for intervention and mortality [11,12].

While large national audits and tertiary-center studies have shaped much of the current evidence, data from district general hospitals remain limited [1,13,14]. It is essential to understand patterns of presentation in these centers and assess how clinical guidelines are implemented [4].

This study aimed to describe the clinical characteristics, endoscopic findings, interventions, and outcomes of patients presenting with AUGIB to a district general hospital in the United Kingdom.

## Materials and methods

Study design and population

This study was a single-center retrospective observational study. It included all inpatients who presented to Queen’s Hospital, Burton, with complaints of hematemesis and/or melena and underwent upper GI endoscopy between January 1 and December 31, 2024.

Inclusion criteria

All adult inpatients (aged 18 years or older) who presented with symptoms suggestive of AUGIB, specifically hematemesis (vomiting of fresh blood or coffee-ground material), melena (black and tarry stools), or both, and who subsequently underwent diagnostic or therapeutic upper GI endoscopy during their hospital admission were included.

Exclusion criteria

Patients were excluded if their endoscopy was performed on an outpatient basis or if electronic medical records were incomplete or inaccessible (e.g., missing key variables such as GBS, endoscopic findings, or outcomes).

Endoscopic procedures

Endoscopic procedures were performed in accordance with the BSG and the ESGE guidelines [9,10]. All procedures were undertaken by GI consultants and trained nurse endoscopists.

Ethical considerations

This study was part of an annual hospital-wide AUGIB audit. Given the retrospective nature of the study and the use of de-identified data, the requirement for informed consent was waived. No identifiable patient information was extracted or reported.

Data collection

Data extraction was performed systematically from two primary sources: the hospital's electronic medical records system and the dedicated endoscopy reporting system (Medilogik). All data were collected by the authors using a predesigned study proforma. Variables collected included age, sex, presentation (hematemesis and/or melena), hemodynamic status (defined as systolic blood pressure <100 mmHg and/or heart rate >100 bpm, or clinical signs of shock requiring fluid resuscitation), pre-endoscopic treatment, endoscopic findings, therapeutic interventions, and outcomes.

Risk stratification was done using the GBS calculated at admission, including components such as blood urea nitrogen, hemoglobin levels, systolic blood pressure, pulse rate, melena, syncope, and presence of comorbidities. Causes of bleeding were classified as nonvariceal or variceal; specific diagnoses (e.g., duodenal ulcer, gastric ulcer, esophagitis, varices, and Mallory-Weiss tear); stigmata of recent hemorrhage (using Forrest classification for nonvariceal bleeds); and variceal grading (e.g., grade 1-3 based on size of varices and risk features) were recorded.

Endoscopic therapies used were classified as single, dual, or triple modalities. Incidence of re-bleeding was defined as recurrent hematemesis, melena, or hemodynamic instability within 30 days; need for repeat endoscopy or referral to tertiary care; and in-hospital mortality.

Statistical analysis

Descriptive statistics were used to summarize patient characteristics, endoscopic findings, and outcomes. Continuous variables (e.g., age) were reported as means ± SD, and categorical variables (e.g., sex and etiology) as frequencies and percentages. No subgroup analyses or multivariable modeling were performed due to the sample size and study objectives. A p-value threshold was not applicable, as the focus was on descriptive statistics rather than comparative inference. All statistical analyses were conducted using IBM Statistical Package for the Social Sciences (SPSS), Version 23 (IBM Corp., Armonk, NY, USA).

## Results

Demographic and clinical characteristics

A total of 64 patients were included, with a mean age of 72.1±15.8 years (range 22-94 years) and a male predominance (71%, n=45). All patients (100%) presented with hematemesis, melena, or both, with suspicion of AUGIB as the indication for endoscopy. In addition to their presenting symptoms, several patients had clinical factors that could influence bleeding risk. Alcohol use was reported by 13 patients (20%), while six patients (9%) were taking NSAIDs at the time of admission. Anticoagulant or antiplatelet therapy was documented in 11 patients (17%). A history of liver disease or cirrhosis was noted in nine patients (14%). These demographic and clinical characteristics are summarized in Table 1.

**Table 1:** Baseline patient demographics and clinical characteristics (N=64) N refers to the total number of patients included in the study cohort. Values are presented as numbers (percentages). Age is reported as mean ± SD. NSAIDs, nonsteroidal anti-inflammatory drugs; GBS, Glasgow-Blatchford Score

Characteristic	Value
Total patients	64
Age, years (mean ± SD)	72.1±15.8
Age range, years	22-94
Sex	Male 45 (71%); female 19 (29%)
Hemodynamic instability on presentation	3 (4.7%)
Presentation symptoms	Hematemesis and/or melena (100%)
Alcohol use	13 (20%)
NSAID use	6 (9%)
Anticoagulant or antiplatelet use	11 (17%)
History of liver disease/cirrhosis	9 (14%)
GBS score calculated on admission	64 (100%)

Risk stratification and hemodynamic status

All patients (100%) had the GBS risk assessment completed at admission or before endoscopy to determine prognosis and timing of intervention. Three patients (4.7%) were hemodynamically unstable at presentation, necessitating urgent resuscitation and subsequent expedited endoscopy.

Endoscopic findings

**Figure 1:**
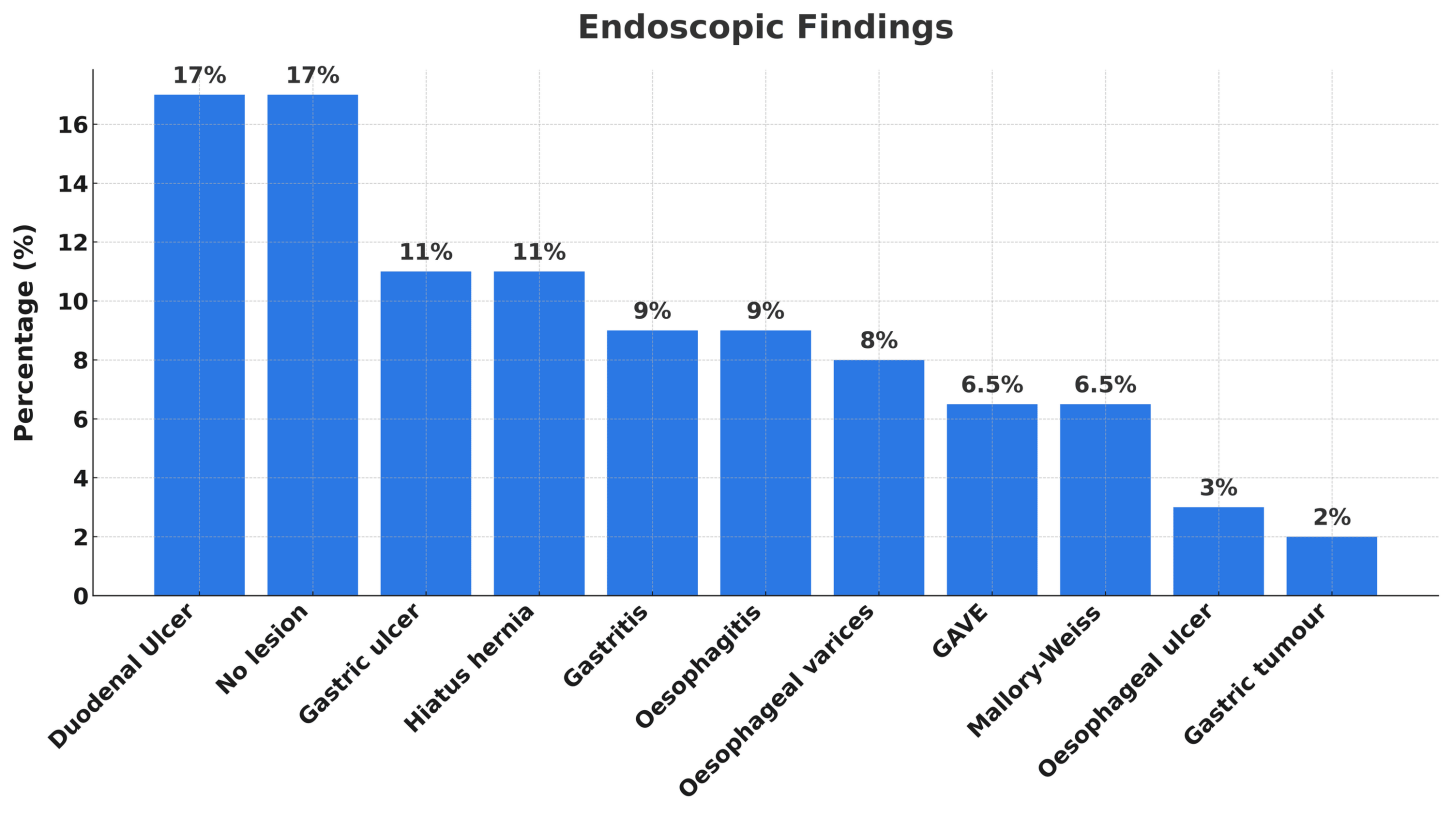
Distribution of endoscopic findings in our study In our study, duodenal ulcer (17%) and no lesion (17%) were the most frequent endoscopic findings, followed by gastric ulcer (11%), hiatal hernia (11%), gastritis (9%), and esophagitis (9%). Less common findings included esophageal varices (8%), GAVE (7%), Mallory-Weiss tear (7%), esophageal ulcer (3%), and gastric tumor (2%). Percentages were rounded to the nearest whole number. GAVE, gastric antral vascular ectasia

Nonvariceal bleeding accounted for 59 patients (92%), while variceal bleeding accounted for five patients (8%). In 11 cases (17%), no bleeding source was visualized. When active or ongoing bleeding was suspected, further investigations such as CT angiography were performed. Other notable endoscopic findings included esophagitis in six patients (9%), gastritis in six (9%), esophageal varices in five (8%), gastric antral vascular ectasia (GAVE) in four (6.5%), Mallory-Weiss tears in four (6.5%), esophageal ulcers in two (3%), and a gastric tumor in one patient (1.6%). Below is a chart showing the endoscopic findings (Figure 1).

Therapeutic interventions

Among nonvariceal bleeds, 15 patients (25%) required endoscopic therapy. Treatments included adrenaline spray, hemoclips, cyanoacrylate glue, thermal modalities, and argon plasma coagulation. Single-modality adrenaline injection alone was used in only 20% of treated cases. Combination therapy was more commonly employed, including dual therapy (53%) and triple therapy (27%).

All patients suspected of having variceal bleeding were commenced on prophylactic antibiotics, indicating very high compliance with recommended standards of care in this group. Early medical optimization was undertaken in all cases, and two patients received terlipressin at admission. During endoscopy, two patients (40%) with high-risk (grade 2 or 3) esophageal varices underwent endoscopic band ligation.

Outcomes

Overall, 66% (42/64) of patients underwent gastroscopy within 24 hours of presentation. The average time to inpatient endoscopy was 1.4 days (range one to three days). Delays were most common among patients presenting out of hours, as the hospital does not provide endoscopy services overnight or on weekends. These patients typically underwent the procedure the following day, although most still met the recommended 24-hour timeframe. Clinical outcomes in this group remained favorable.

Although the exact length of hospital stay was not recorded, later endoscopy, particularly among those presenting out of hours, may have contributed to longer admissions for some patients due to extended monitoring while awaiting the procedure. Re-bleeding occurred in two patients (3.1%). Both were transferred to a tertiary center for further endoscopic or radiological intervention, and escalation was carried out promptly through established referral pathways. All other patients were discharged safely, and no in-hospital deaths occurred during the study period.

## Discussion

This study provides important insights into the clinical characteristics, endoscopic findings, and outcomes of patients presenting with AUGIB in a district general hospital. Our findings indicate that AUGIB predominantly affects older adults (mean age 72 years) and occurs more frequently in males [1,3]. This demographic pattern is consistent with national and international data, which identify advanced age and male sex as established risk factors for AUGIB [1,3,7]. The association with older age may reflect the higher prevalence of cardiovascular comorbidities, polypharmacy, and increased use of NSAIDs and anticoagulant therapies in this population [4,15]. These similarities demonstrate that our local patient population reflects epidemiological trends seen in previous United Kingdom and European studies.

Endoscopic findings

In our cohort, nonvariceal bleeding accounted for 92% of cases, with duodenal ulcers (17%) and gastric ulcers (11%) being the most common etiologies. This predominance of peptic ulcer disease aligns with earlier United Kingdom audits and European series, despite the declining prevalence of Helicobacter pylori infection [1,4,16]. These findings highlight the continued clinical burden of ulcer-related bleeding, particularly among patients exposed to antithrombotic agents [4], and suggest that the underlying causes of AUGIB in our hospital are comparable to those reported in larger national datasets.

Seventeen percent of patients had no identifiable source of bleeding at endoscopy. Similar rates (10-20%) have been reported in other studies [15,17,18]. Possible explanations include spontaneous cessation of bleeding before endoscopy, intermittent hemorrhage from vascular lesions such as angiodysplasia, or lesions missed due to suboptimal visualization [19-21]. Although these patients generally have favorable outcomes, they require close monitoring for the recurrence of symptoms [20,21]. Our approach to investigating and monitoring these cases is consistent with recognized strategies described in previous studies.

Variceal bleeding was relatively uncommon (8%) in our cohort, consistent with national trends for AUGIB [1,6]. Despite its low frequency, variceal hemorrhage remains clinically significant because of its higher mortality and re-bleeding risk [6].

Therapeutic interventions

Approximately one quarter of patients with nonvariceal bleeding required therapeutic endoscopy, with dual or triple therapy being the most frequently employed modalities [22]. Evidence strongly supports the use of combination therapy, involving adrenaline injection followed by thermal or mechanical hemostasis, over monotherapy in reducing re-bleeding rates [18,22]. Our findings reinforce this practice, and the predominance of multimodal therapy in our unit indicates that our therapeutic approach aligns with current guideline recommendations for the management of nonvariceal bleeding.

The use of prophylactic antibiotics, terlipressin, and endoscopic band ligation for variceal bleeding followed established guidelines [6]. Antibiotics reduced the occurrence of complications as well as mortality associated with cirrhosis, while appropriate endoscopic therapeutic interventions reduced the incidence of re-bleeding and mortality [6]. All patients with suspected variceal bleeding received prophylactic antibiotics, indicating very high compliance with recommended standards of care in this group. This consistent application of evidence-based therapy reflects robust local practice in managing these high-risk patients and demonstrates that local management of variceal bleeding reflects recognized best practice standards.

Outcomes

The re-bleeding rate in this study was low (3.1%), which is lower than the 7-30% reported in the literature [1,23]. Several factors likely contributed to this favorable outcome, including systematic use of the GBS, appropriate triaging, and timely endoscopy within recommended timeframes [7,11]. Most patients underwent endoscopy within 24 hours of arrival, and this timely access to investigation appears to have played an important role in achieving low re-bleeding rates and preventing in-hospital mortality. No deaths occurred in our cohort, a finding that compares favorably with national data showing mortality rates of 7-10% [1,2].

The proportion of patients requiring endoscopic therapy was relatively small, suggesting that many presented with less severe bleeding. This reinforces the usefulness of structured risk assessment at an early stage in determining which patients may safely avoid intervention.

Although patients presenting out of hours experienced some delay due to the absence of overnight and weekend endoscopy services, most still received their procedures within an acceptable timeframe. These delays may have extended hospital stay for certain individuals, but overall clinical outcomes remained reassuring.

Limitations

Limitations of this study include its retrospective design, modest sample size, and lack of long-term follow-up data on re-bleeding and mortality. Future research should focus on multicenter collaborations to generate larger datasets, examine variations in clinical practice, and explore the impact of different therapeutic approaches on patient outcomes.

## Conclusions

This study demonstrates that AUGIB in a district general hospital predominantly affects elderly male patients and is most commonly caused by nonvariceal etiologies, particularly peptic ulcer disease. Routine risk stratification using the GBS, timely endoscopy, and evidence-based therapeutic interventions were associated with favorable outcomes, including a low re-bleeding rate and absence of in-hospital mortality. The ability to provide early endoscopy for most patients, even with limited out-of-hours services, likely contributed to these positive outcomes and highlights the importance of prompt assessment and treatment in managing AUGIB. These results are consistent with findings from other centers and show that high-quality care for AUGIB can be delivered effectively in a district hospital setting.
